# THE IMPACT OF A FACILITATED DISCUSSION SERIES IN IMPROVING DEMENTIA CAREGIVER STRESS, RESILIENCY, AND WELL-BEING

**DOI:** 10.1093/geroni/igac059.1765

**Published:** 2022-12-20

**Authors:** Robin Bonifas, Wendy Cohen

**Affiliations:** Indiana State University, Terre Haute, Indiana, United States; Duet: Partners in Health & Aging, PHOENIX, Arizona, United States

## Abstract

Emotional stress stemming from the complex grief associated with dementia care takes a greater toll on a family caregiver’s health than the physical effort of providing care. Finding Meaning and Hope, a 10-week workshop for caregivers of persons with dementia designed to promote understanding of grief and teach self-care concepts that mitigate stress. Each session features a video of a dementia care expert sharing strategies, followed by a facilitator-led discussion. Home practice activities help caregivers incorporate focal self-care strategies into their lives. This evaluation research assessed the association between workshop participation and caregiver outcomes in stress and well-being. 208 caregivers, 83 percent age 60 and older, completed the workshops between 2019 and 2021. Two standardized instruments were administered before and after workshop participation: the 12-item Caregiver Self-Assessment Questionnaire (American Medical Association, 2015) and the 11-item Sense of Competence Questionnaire (Vernooij-Dassen, 2008). Multivariate Linear Regression was used to assess factors associated with participant outcomes. Participation in more than half the sessions had a positive impact on stress management, emotional well-being, and coping, but fewer gains were made by caregivers with health limitations and those who began the workshops with lower emotional well-being ( F = 15.923, p < .001). Given that family caregivers’ mental and emotional health has been negatively impacted by the COVID-19 pandemic (Czeisler et al. 2020), strategies to manage stress and bolster well-being are vital in enabling them to continue care provision when isolation may exacerbate the challenges they encounter. Finding Meaning and Hope offers a promising approach.

